# GSK2126458 has the potential to inhibit the proliferation of pancreatic cancer uncovered by bioinformatics analysis and pharmacological experiments

**DOI:** 10.1186/s12967-021-03050-7

**Published:** 2021-08-30

**Authors:** Yueqin Feng, Yuguan Jiang, Fengjin Hao

**Affiliations:** 1 Department of Ultrasound, The First Affiliated Hospital of China Medical University, Shenyang, 110022 Liaoning China; 2 School of Pharmacy, China Medical University, Shenyang, 110122 Liaoning China; 3 Department of Biochemistry and Molecular Biology, China Medical University, Shenyang, 110122 Liaoning China

**Keywords:** Pancreatic cancer, Bioinformatics analysis, GEO, DAVID, PI3K–Akt

## Abstract

**Background:**

Pancreatic cancer is one of the most serious digestive malignancies. At present, there is an extreme lack of effective strategies in clinical treatment. The purpose of this study is to identify key genes and pathways in the development of pancreatic cancer and provide targets for the treatment of pancreatic cancer.

**Methods:**

GSE15471 and GSE62165 were used to screen differentially expressed genes by GEO2R tool. Hub genes prognostic potential assessed using the GEPIA and Kaplan–Meier plotter databases. The drug susceptibility data of pan-cancer cell lines is provided by The Genomics of Drug Sensitivity in Cancer Project (GDSC). Finally, the effects of PI3K–Akt signaling pathway inhibitors on cell viability of pancreatic cancer cells were detected by cell proliferation and invasion assays.

**Results:**

A total of 609 differentially expressed genes were screened and enriched in the focal adhesion, phagosome and PI3K–Akt signaling pathway. Of the 15 hub genes we found, four were primarily associated with the PI3K–Akt signaling pathway, including COL3A1, EGF, FN1 and ITGA2. GDSC analysis showed that mTOR inhibitors are very sensitive to pancreatic cancer cells with mutations in EWSR1.FLI1 and RNF43. Cell proliferation and invasion results showed that mTOR inhibitors (GSK2126458) can inhibit the proliferation of pancreatic cancer cells.

**Conclusions:**

This study suggested that the PI3K–Akt signaling pathway may be a key pathway for pancreatic cancer, our study uncovered the potential therapeutic potential of GSK2126458, a specific mTOR inhibitor, for pancreatic cancer.

**Supplementary Information:**

The online version contains supplementary material available at 10.1186/s12967-021-03050-7.

## Background

Pancreatic cancer is one of the most devastating and frequent malignancies worldwide, and its incidence rate is still nearly equal to its death rate. It’s the fourth leading cause of cancer-related death globally and is associated with the lowest 5-year survival rate of less than 5% known for human cancers [1–4]. Although great progress in the treatment of pancreatic cancer, it is still one of the leading causes of death. The main cause of high mortality in pancreatic cancer is that pancreatic cancer has the biological features that other solid tumors do not have: abnormal tumor metabolism, the high degree of cell malignancy, lack of blood supply to tumor cells and complex tumor microenvironment. Most tumor growth processes need to generate a large number of blood vessels to provide nutrition [5, 6]. However, pancreatic cancer is not sensitive to nutrient supply and pancreatic cancer can still be growing rapidly in a nutrient-deficient and sparsely-blood-driven environment [7, 8]. So far, although more and more evidence suggested that multiple genes and cellular pathways are involved in the development and progression of pancreatic cancer, the lack of knowledge about the exact molecular mechanisms underlying pancreatic cancer progression has limited the ability to treat the diseases. Therefore, identifying the hub genes (genes that play a vital role in biological processes) and key pathways of this disease is important for further studying the pathogenesis of pancreatic cancer and develop more effective treatment methods.

Gene chip, a gene detection technology, can quickly detect all the gene expression information at the same time point [9]. Nowadays, high throughput sequencing is becoming more widely used and has been used as a very effective tool in life science, such as early diagnosis of cancer, classification of tumor and prognosis prediction [10]. Therefore, a large number of gene profile data have been produced with the widespread use of gene chips, and most of the data has been stored in public databases. Integrating and re-analyzing these data can provide valuable clues for new research, which is especially suitable for the screening of differentially expressed genes (DEGs). Hence, the integrated bioinformatics methods were used to explore the pathogenesis of pancreatic cancer. Bioinformatics analysis studies have identified several potential biomarkers [11–14]. However, these results do not adequately explain how pancreatic cancer cells survive hypoxic conditions, which are often associated with inadequate nutrient supply.

In this work, we identified DEGs in pancreatic cancer using bioinformatics methods. The original data GSE15471 and GSE62165 were chosen from Gene Expression Omnibus (GEO), and DEGs were filtered by the GEO2R online tool. GSE15471 and GSE62165 have reasonable experimental design and reliable data quality, which can provide rich information for data mining [11, 13, 15–17]. Followed by, Gene ontology (GO) and pathway enrichment analysis were performed with DAVID [18, 19]. Moreover, we constructed protein–protein interaction (PPI) network of the DEGs and modular analysis to pick out hub genes in pancreatic cancer. Then we used GEPIA to validate the expression of hub genes between cancer patients and healthy people. Finally, the effects of PI3K–Akt signaling pathway inhibitors (mTOR inhibitor, GSK2126458) on cell viability of pancreatic cancer cells were detected by MTT, colony and invasion assays. The analysis of DEGs’ biological functions and pathways will provide better insight into molecular mechanism and potential candidate therapeutic targets for pancreatic cancer.

## Materials and methods

### Cell lines and reagents

The human pancreatic cancer cell lines (PANC-1) were purchased from the Chinese Academy of Sciences Cell Bank and cultured in DMEM medium with 10%FBS, 100 U/mL of penicillin, 100 μg/mL of streptomycin. The cells were cultured in a 5% CO_2_ incubator at 37 ℃. GSK2126458 was purchased from ApexBio (USA).

### MTT assay

Logarithmic growth phase cells were seeded in 96-well plates. After 24 h, the cells cultured in different concentrations of GSK2126458 (1.0 μmol/L, 0.5 μmol/L, 0.25 μmol/L) for 24 h, 48 h or 72 h. MTT (final concentration: 5 mg/mL) were added to each well and then incubated for 4 h in the incubator. We discarded the culture solution and added 150 μL of DMSO (dimethyl sulfoxide) to each well, and measured the absorbance at 490 nm after shaking.

$$\text{Cell viability} (\%)=({\text{OD}_{\text{sample}}}-{\text{OD}_{\text{blank}}})/({\text{OD}_{\text{control}}} - {\text{OD}_{\text{blank}}}) * 100 \%.$$

### Microarray data information

The gene expression profiles of GSE15471 and GSE62165 were obtained from the GEO database (https://www.ncbi.nlm.nih.gov/geo). The microarray data of GSE15471 was based on GPL570 Platforms ([HG-U133_Plus_2] Affymetrix Human Genome U133 Plus 2.0 Array) and included 39 pancreatic cancer tissues and 39 normal pancreatic tissues (Submission date: Mar 31, 2009) [20]. GSE15471 contain “pairs” of normal pancreatic tissues and pancreatic cancer tissues samples. The microarray data of GSE62165 was based on GPL13667 Platforms ([HG-U219] Affymetrix Human Genome U219 Array) and included 118 pancreatic cancer tissues and 13 normal pancreatic tissues (Submission date: Oct 08, 2014) [21]. The two gene expression profiles obtained from pancreatic ductal adenocarcinoma (PDAC). GEO2R (https://www.ncbi.nlm.nih.gov/geo/geo2r/) will give a box plot to determine if selected samples are suitable for comparison. Viewing the distribution is important for determining if the selected samples are suitable for comparison; Generally, median-centered values are indicative that the data are normalized and cross-comparable.

### DEGs identification

GEO2R (https://www.ncbi.nlm.nih.gov/geo/geo2r/) is an interactive web tool for detecting DEGs [22]. GEO2R allows users to compare two or more groups of samples to analyze most of the GEO series with gene symbol. We used GEO2R to identify genes that are differentially expressed between pancreatic cancer samples and normal samples. GEO2R is data processed by ebayes algorithm in limma package in R language [22]. The adjust P-value < 0.05 and |logFC|> 1 were set as cut-off criteria. We used log2-fold change between two groups. Then the co-expression up-regulated and down-regulated genes of the two expression profiles were identified in the Venn diagram (http://bioinformatics.psb.ugent.be/webtools/Venn/). The heat map was plotted for samples and top 50 DEGs in Heatmao IIIustrator software (Heml 1.0.3.7) [23].

### GO function and KEGG pathway enrichment analysis of DEGs

GO analysis is a useful method for annotating genes and gene products and for identifying the characteristic biological properties of high throughput genome or transcriptional data [24, 25]. KEGG is a database including biological pathway, diseases, drugs, and chemicals [26]. The Database for Annotation, Visualization and Integrated Discovery (DAVID, https://david.ncifcrf.gov/), a functional annotation tool, is a website which can provide GO analysis and pathway analysis. These analyses were performed using DAVID online tool to analyze the DEGs at the functional level. P-value < 0.05 was set as the cut-off criterion.

### The construction of PPI network, module analysis and significant candidate genes and pathway identification

In order to study the protein–protein interaction (PPI) information, Search Tool for the Retrieval of Interacting Genes (STRING, version 10.5, https://string-db.org/) database was used and combined score > 0.4 was selected as cut-off criteria [27]. Subsequently, the PPI networks were built and visualized in Cytoscape software (version 3.6.0) [28]. The Molecular Complex Detection (MCODE) was used to analyze the modules of the PPI network with the default parameters settings such as degree cutoff = 2, node score cutoff = 0.2, k-core = 2, max. depth = 100. The cut-off criteria were set as follows: MCODE score > 4 and number of nodes > 4. Moreover, the function and pathway enrichment analysis of genes in the modules were performed by STRING.

### Comparison of the hub genes expression level

The GEPIA (http://gepia.cancer-pku.cn/index.html) is a newly developed online server for interaction analysis. The standard processing pipeline is used to analyze the RNA sequence expression data of 9736 tumor samples and the 8587 normal samples from the TCGA (The Cancer Genome Atlas) and the GTEx (Genotype-Tissue Expression) projects [29]. TCGA database can compare the differential expression analysis between normal tissue and tumor tissue. However, due to the fact that its normal samples are also from cancer patients, the clustering of samples may be confused. The normal sample data of GTEx are from health people. Considering that there are too few normal samples in TCGA, the data of above two databases are merged on GEPIA website for analysis. It can provide custom functions for tumor and normal differential expression analysis. We used the boxplot to display the expression of hub genes in pancreatic cancer tissues and normal tissues. This study took the P-value less than 0.01 as the cutoff point. Furthermore, the human protein Atlas (HPA) shows the protein level of 4 hub genes in the PI3K–Akt pathway in pancreatic cancer and normal tissues [30, 31].

### Survival curve analysis

Kaplan–Meier plotter was used to analyze the RNA-seq data in TCGA, EGA and geo databases (http://kmplot.com/analysis/). It can evaluate the effect of more than 54,000 biomolecules on the survival rate of various tumor tissues. Here, the Kaplan Meier plotter was used to analyze the association between key genes (COL3A1, FN1 and ITGA2) expression and survival in patients with pancreatic cancer. We searched the database with COL3A1, FN1 and ITGA2 as the input and analyzed all the samples in the database. The parameters are as previously reported [32]: cut-off value of grouping: median; hazard ratio: Yes; 95% confidence interval: Yes.

### Chemotherapeutic response

We analyzed the largest open pharmacogenomics database to predict the chemotherapeutic response of PI3K–Akt–mTOR specific inhibitors (PI3K: BKM-120, Akt: MK-1102, and mTOR inhibitor: GSK2126458) to each sample [cancer drug sensitivity genomics (GDSG)], https://www.cancerrxgene.org/]. The analysis method is based on the previous reports [32].

### Cell culture

PNAC-1 cells and BxPC-3 cells (purchased from Procell, China) in RPMI-1640 medium supplemented with 10% fetal bovine serum (FBS) and 1% penicillin–streptomycin, at 37 °C, 5% Incubate in CO_2_ gas phase.

### Cell viability

PNAC-1 cells and BxPC-3 cells were seeded in a 96-well plate, cultured overnight in an incubator, and treated with or without GSK2126458 (0–10 μM) for 24 h, 48 h, and 72 h. Then, the medium was added to the 5 mg/mL (final concentration) MTT solution and incubated for 4 h. The formed formazan crystals were dissolved in 150 μL DMSO per well and measured at 490 nm by a microplate reader (Elx800 Bio-Tek, USA). Repeat the experiment at least 3 times.

### Colony formation

The cells were seeded on 6-well plates, cultured overnight, and treated with different concentrations of DMSO or GSK2126458 for 48 h. Then washed with PBS and cultured in complete growth medium for another 10 days. The fresh medium was changed every 3 days. The cells were fixed with 100% methanol and stained with 0.1% crystal violet.

### Invasion assay

The experimental operation was carried out according to the method previously reported [33]. PANC-1 cells in logarithmic growth phase were seeded into the upper layer of 8 μm transwell chamber. The diluted matrix glue is added to the upper layer of the chamber in advance for solidification. After 48 h of culture with different concentrations of GSK2126458 (0–1 μmol/L), PANC1 cells invading the subventricular layer were stained with 0.1% crystal violet. The invasive cells were observed and photographed under an inverted microscope (OLYMPUS, IX71).

### FACS assay

The experimental methods refer to the previous literature [34]. In general, PANC-1 cells were treated with different concentrations of GSK2126458 for 48 h, and the cells were digested with trypsin to obtain cell suspension. The cells were centrifuged at 4 °C and 3000 rpm for 10 min, then completely resuspended and washed. The cells were then centrifuged at 4 °C and 2000 rpm for 10 min. We discarded the supernatant, added binding buffer, annexin V-FITC and PI working solution (BD pharmingen, USA) to each sample, mixed them evenly, and then filtered them with a 400 mesh filter to obtain single cell suspension. The number of apoptotic cells was detected by flow cytometry (BD, C6).

### Quantitative PCR

Total RNA of PNAC-1 cells after treated with GSK2126458 for 48 h was extracted using TRIzol reagent (Qiagen, USA), and cDNA was synthesized from total RNA (2 μg) using the first-strand synthesis system (Vazyme, China). Dilute the cDNA to 2 ng/µL, then add 4 µL to 10 µL 2×  FastStart Universal SYBR Green PCR Master (Vazyme, China). Each sample was tested in triplicate using the StepOnePlus qPCR system (Applied Biosystems 7500). The Ct value was normalized to the housekeeping gene GAPDH, which was amplified in parallel. The following qPCR primers were used: COL3A1-F, 5′-GGAGCTGGCTACTTCTCGC-3′ and COL3A1-R, 5′-GGGAACATCCTCCTTCAACAG-3′; EGF-F, 5′-TGGATGTGCTTGATAAGCGG-3′ and EGF-R, 5′-ACCATGTCCTTTCCAGTGTGT-3′; FN1-F, 5′-CGGTGGCTGTCAGTCAAAG-3′ and FN1-R, 5′-AAACCTCGGCTTCCTCCATAA-3′; ITGA2-F, 5′-CCTACAATGTTGGTCTCCCAGA-3′ and ITGA2-R, 5′-AGTAACCAGTTGCCTTTTGGATT-3′; GAPDH-F, 5′-TGGTGTCTGAGGGTTCTGTGG-3′ and GAPDH-R, 5′- TGATGACCCTTTTGGCTCCC-3′.

### Statistical analysis

All data were represented by mean ± SD. Significant differences were calculated in GraphPad 5.0 (Inc., La Jolla, CA, USA) with one-way ANOVA analysis, and then Student t-test or Tukey’s multiple comparison test was conducted. *P* < 0.05 was significant difference.

## Results

### Identification of DEGs

**Fig. 1:**
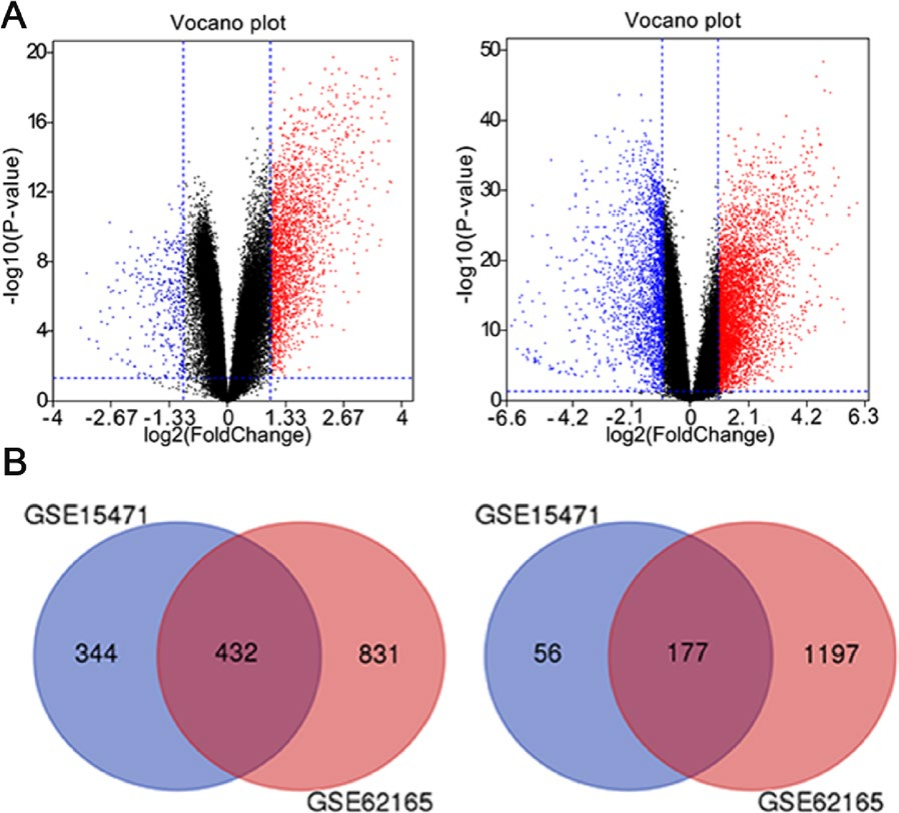
**A** Volcano plot of differentially expressed genes in GSE15471 (left; scale bar = 1.33) and GSE62165 (right; scale bar = 2.1). Red: up-regulated genes; blue: down-regulated genes; **B** Identification of co-expression of up-regulated (left) and down-regulated (right) genes in GSE15471 and GSE62165

GSE15471 and GSE62165 were downloaded free from GEO database. Due to various reasons such as background and probe design, the original microarray data leads to huge differences between the microarray data. Therefore, it is necessary to check whether the data is highly standardized gene expression profile data, which can be used for subsequent DEGs analysis (Additional file 1: Figure S1). Then the GEO2R online analysis tool was used to detect differentially expressed genes. The screening criteria were adjust P-value < 0.01 and |logFC|> 1. The volcano plot (Fig. 1) showed the distribution of DEGs in GSE15471 and GSE62165. A total of 776 up-regulated DEGs were identified with the 233 down-regulated DEGs from GSE15471. And a total of 1263 up-regulated DEGs were identified with the 1374 down-regulated DEGs from GSE62165. Heat maps of the top 50 differentially expressed genes (30 up-regulated and 20 down-regulated) of GSE15471 and GSE62165 were all shown in Additional file 1: Figure S2. After a comprehensive comparative analysis, 609 differentially expressed genes were identified, including 432 up-regulated genes and 177 down-regulated genes in pancreatic cancer tissues compared with normal pancreatic tissues in two expression datasets (Additional file 1: Table S1). As shown in Fig. 1, the corresponding Venn diagrams showed the overlap region of DEGs in two gene expression profiles.

### GO function enrichment analysis

**Fig. 2:**
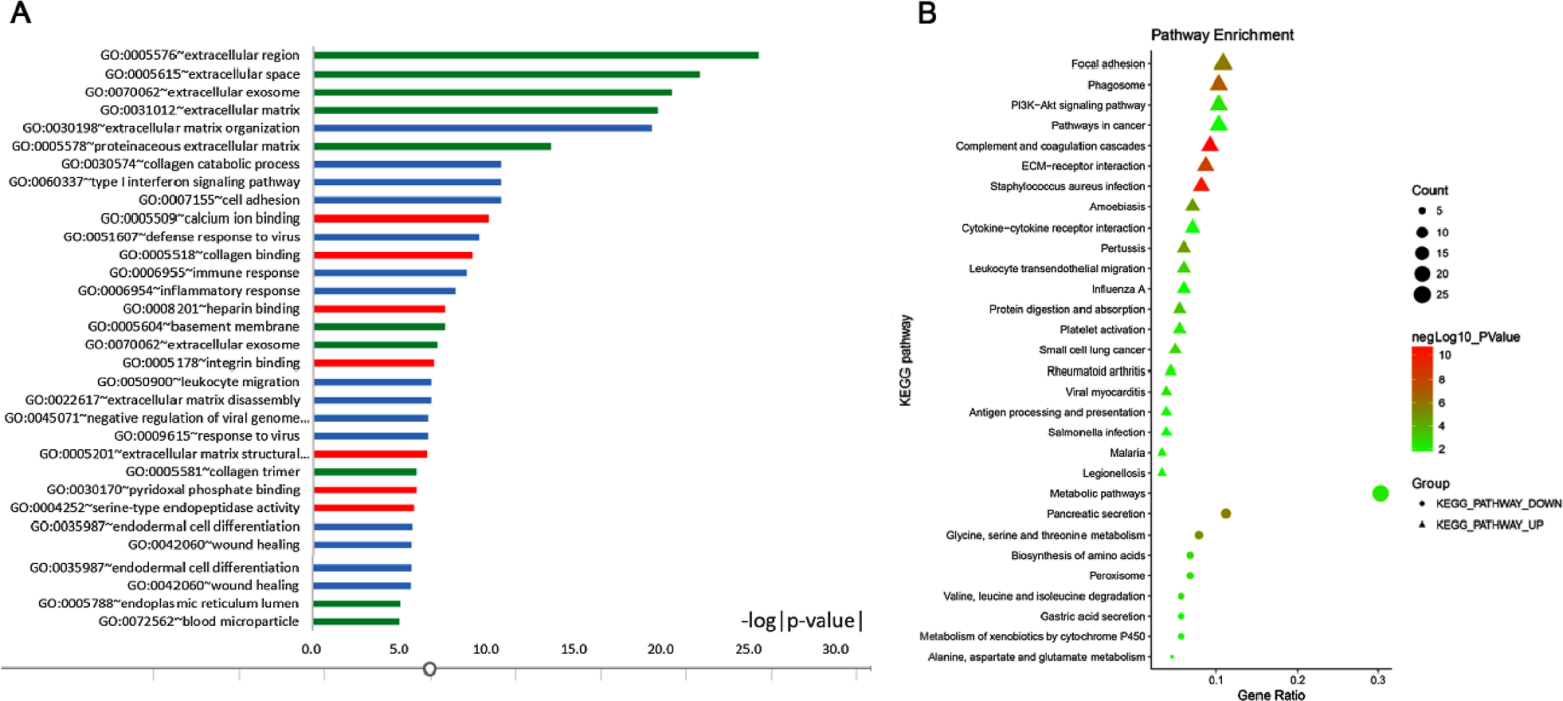
The significant enriched go terms and KEGG pathway (top 30) of DEGs in pancreatic cancer

We submitted the 609 DEGs to the online database DAVID for Gene ontology analysis in pancreatic cancers. In this study, the total of 253 enriched GO terms have been identified with the criteria P-value < 0.05, and the top 15 enriched GO terms of up-regulated DEGs and down-regulated DEGs were listed in Table 1, respectively. The significantly enriched go terms (top 30) of DEGs in pancreatic cancer were shown in Fig. 2A. The GO analysis results showed that DEGs were divided into three functional groups: biological processes (BP), molecular function (MF) and cell component (CC). To summarize, these results showed that most of the DEGs were mainly enriched in metabolic process, binding and extracellular part.

**Table 1:** Gene ontology analysis of differentially expressed genes associated with pancreatic cancer

Expression	Category	Term	Gene count	%	P value
Up-regulated	GOTERM_BP_DIRECT	GO:0030198 ~ extracellular matrix organization	35	8.10	4.85E−20
	GOTERM_BP_DIRECT	GO:0060337 ~ type I interferon signaling pathway	16	3.70	1.72E−11
	GOTERM_BP_DIRECT	GO:0030574 ~ collagen catabolic process	16	3.70	1.72E−11
	GOTERM_BP_DIRECT	GO:0007155 ~ cell adhesion	39	9.03	1.88E−11
	GOTERM_BP_DIRECT	GO:0051607 ~ defense response to virus	22	5.09	3.53E−10
	GOTERM_MF_DIRECT	GO:0005509 ~ calcium ion binding	48	11.11	9.05E−11
	GOTERM_MF_DIRECT	GO:0005518 ~ collagen binding	14	3.24	7.95E−10
	GOTERM_MF_DIRECT	GO:0008201 ~ heparin binding	19	4.40	3.00E−08
	GOTERM_MF_DIRECT	GO:0005178 ~ integrin binding	15	3.47	1.25E−07
	GOTERM_MF_DIRECT	GO:0005201 ~ extracellular matrix structural constituent	12	2.78	3.33E−07
	GOTERM_CC_DIRECT	GO:0005576 ~ extracellular region	111	25.69	3.52E−26
	GOTERM_CC_DIRECT	GO:0005615 ~ extracellular space	95	21.99	7.82E−23
	GOTERM_CC_DIRECT	GO:0070062 ~ extracellular exosome	143	33.10	2.98E−21
	GOTERM_CC_DIRECT	GO:0031012 ~ extracellular matrix	42	9.72	1.88E−20
	GOTERM_CC_DIRECT	GO:0005578 ~ proteinaceous extracellular matrix	33	7.64	2.75E−14
Down-regulated	GOTERM_BP_DIRECT	GO:0072593 ~ reactive oxygen species metabolic process	6	3.39	1.84E−05
	GOTERM_BP_DIRECT	GO:0008652 ~ cellular amino acid biosynthetic process	5	2.82	9.84E−05
	GOTERM_BP_DIRECT	GO:0006520 ~ cellular amino acid metabolic process	5	2.82	5.42E−04
	GOTERM_BP_DIRECT	GO:0071294 ~ cellular response to zinc ion	4	2.26	7.22E−04
	GOTERM_BP_DIRECT	GO:0006508 ~ proteolysis	14	7.91	9.58E−04
	GOTERM_MF_DIRECT	GO:0030170 ~ pyridoxal phosphate binding	8	4.52	1.43E−06
	GOTERM_MF_DIRECT	GO:0005385 ~ zinc ion transmembrane transporter activity	4	2.26	1.28E−03
	GOTERM_MF_DIRECT	GO:0019899 ~ enzyme binding	11	6.21	1.80E−03
	GOTERM_MF_DIRECT	GO:0004435 ~ phosphatidylinositol phospholipase C activity	4	2.26	2.34E−03
	GOTERM_MF_DIRECT	GO:0042803 ~ protein homodimerization activity	17	9.60	2.43E−03
	GOTERM_CC_DIRECT	GO:0070062 ~ extracellular exosome	55	31.07	8.36E−08
	GOTERM_CC_DIRECT	GO:0005615 ~ extracellular space	29	16.38	5.61E−05
	GOTERM_CC_DIRECT	GO:0016323 ~ basolateral plasma membrane	8	4.52	1.57E−03
	GOTERM_CC_DIRECT	GO:0005887 ~ integral component of plasma membrane	25	14.12	3.38E−03
	GOTERM_CC_DIRECT	GO:0005576 ~ extracellular region	27	15.25	4.39E−03

### KEGG pathway enrichment analysis

The analysis of KEGG pathway enrichment was also using the DAVID online analysis tool. In this study, a total of 40 KEGG pathways have been identified with the criteria P value < 0.05. The significantly enriched KEGG pathways (top 30) of DEGs in pancreatic cancer were shown in Fig. 2B. Table 2 showed the top 15 enriched KEGG pathway of up-regulated DEGs and top 5 enriched KEGG pathway of down-regulated DEGs. As shown in Table 2, the KEGG pathway enrichment analysis of the DEGs showed that the up-regulated DEGs were most significantly enriched in complement and coagulation cascades; the down-regulated DEGs were most significantly enriched in pancreatic secretion glycine. Considering the count of genes, the top three enriched KEGG pathway are focal adhesion, phagosome and PI3K–Akt signaling pathway based on the count of genes (Table 2).

**Table 2:** KEGG pathway analysis of differentially expressed genes associated with pancreatic cancer

Pathway ID		Name	Count	%	P value	Benjamini P value	Genes	Total number^a^
Up-regulated								
hsa04610		Complement and coagulation cascades	17	3.94	1.71E−11	3.17E−09	PLAT, C1QA, C3AR1, C1QB, CD55, FGG, C5AR1, C3, F3, SERPINE1, CFH, C1R, C1S, PROS1, C1QC, PLAU, F2R	78
hsa05150		*Staphylococcus aureus* infection	15	3.47	6.53E−11	6.07E−09	C3AR1, C5AR1, HLA-DRB1, C3, FPR1, C1R, ITGB2, C1S, HLA-DMA, C1QC, C1QA, C1QB, FGG, CFH, FCGR3B	39
hsa04512		ECM-receptor interaction	16	3.7	6.19E−09	3.84E−07	COL4A2, COL4A1, TNC, COL3A1, ITGA2, ITGA3, COL5A1, LAMB3, SDC1, COMP, COL1A2, LAMC2, THBS2, COL11A1, FN1, THBS4	60
hsa04145		Phagosome	19	4.4	9.18E−08	4.27E−06	ACTB, HLA-DRB1, NCF2, C3, ITGA2, C1R, COLEC12, ITGB2, HLA-B, CTSS, HLA-DMA, COMP, TAP1, TUBA4A, THBS2, FCGR3B, CD14, TUBA1C, THBS4	95
hsa04510		Focal adhesion	20	4.63	1.84E−06	6.83E−05	ACTB, COL4A2, COL4A1, TNC, COL3A1, ITGA2, ITGA3, FLNA, COL5A1, VEGFC, LAMB3, RAC2, COMP, COL1A2, LAMC2, PDGFC, THBS2, COL11A1, FN1, THBS4	100
hsa05146		Amoebiasis	13	3.01	2.08E−05	6.45E−04	COL4A2, LAMB3, COL4A1, COL3A1, COL1A2, SERPINB2, LAMC2, ITGB2, SERPINB3, COL11A1, CD14, COL5A1, FN1	69
hsa05133		Pertussis	11	2.55	2.47E−05	6.57E−04	C1QA, C1QB, GNAI1, C3, LY96, PYCARD, ITGB2, C1R, C1S, C1QC, CD14	58
hsa04974		Protein digestion and absorption	10	2.31	4.96E−04	0.011	KCNN4, COL4A2, COL4A1, ATP1B3, COL3A1, COL1A2, COL11A1, COL5A1, COL10A1, SLC7A7	56
hsa04670		Leukocyte transendothelial migration	11	2.55	1.09E−03	0.022	ACTB, CLDN18, RAC2, NCF2, GNAI1, CXCR4, MMP9, CLDN2, ITGB2, RHOH, THY1	75
hsa05222		Small cell lung cancer	9	2.08	1.75E−03	0.032	COL4A2, E2F3, LAMB3, COL4A1, CKS2, ITGA2, LAMC2, ITGA3, FN1	74
hsa04151		PI3K–Akt signaling pathway	19	4.4	4.38E−03	0.066	COL4A2, COL4A1, TNC, COL3A1, ITGA2, ITGA3, IL7R, COL5A1, VEGFC, LAMB3, COMP, COL1A2, LAMC2, PDGFC, THBS2, COL11A1, FN1, THBS4, F2R	99
hsa04611		Platelet activation	10	2.31	7.40E−03	0.101	ACTB, FGG, GNAI1, COL3A1, COL1A2, FCER1G, ITGA2, COL11A1, COL5A1, F2R	89
hsa05323		Rheumatoid arthritis	8	1.85	8.51E−03	0.107	CTSK, TNFSF13B, HLA-DRB1, CCL20, IL18, ITGB2, HLA-DMA, MMP1	63
hsa05144		Malaria	6	1.39	9.16E−03	0.108	SDC1, IL18, COMP, ITGB2, THBS2, THBS4	44
Down-regulated								
hsa04972	Pancreatic secretion		10	5.65	2.34E−06	3.78E−04	PNLIPRP1, PNLIPRP2, CHRM3, RAB3D, PRSS3, CPA2, CELA2B, PLCB1, SLC4A4, CTRL	67
hsa00260		Glycine, serine and threonine metabolism	7	3.95	8.37E−06	6.77E−04	CTH, GATM, GCAT, GAMT, GNMT, PSAT1, CBS	32
hsa01230		Biosynthesis of amino acids	6	3.39	2.41E−03	0.122	BCAT1, CTH, MAT1A, PSAT1, GPT2, CBS	–
hsa00280		Valine, leucine and isoleucine degradation	5	2.82	2.89E−03	0.110	BCAT1, ALDH6A1, AOX1, ABAT, ACAT1	41
hsa04146		Peroxisome	6	3.39	3.98E−03	0.121	EPHX2, PXMP2, CRAT, PEX5L, SLC27A2, PEX7	71

^a^Total number: the number of genes in pathway

### Hub genes and pathways screening from PPI network and modular analysis

**Fig. 3:**
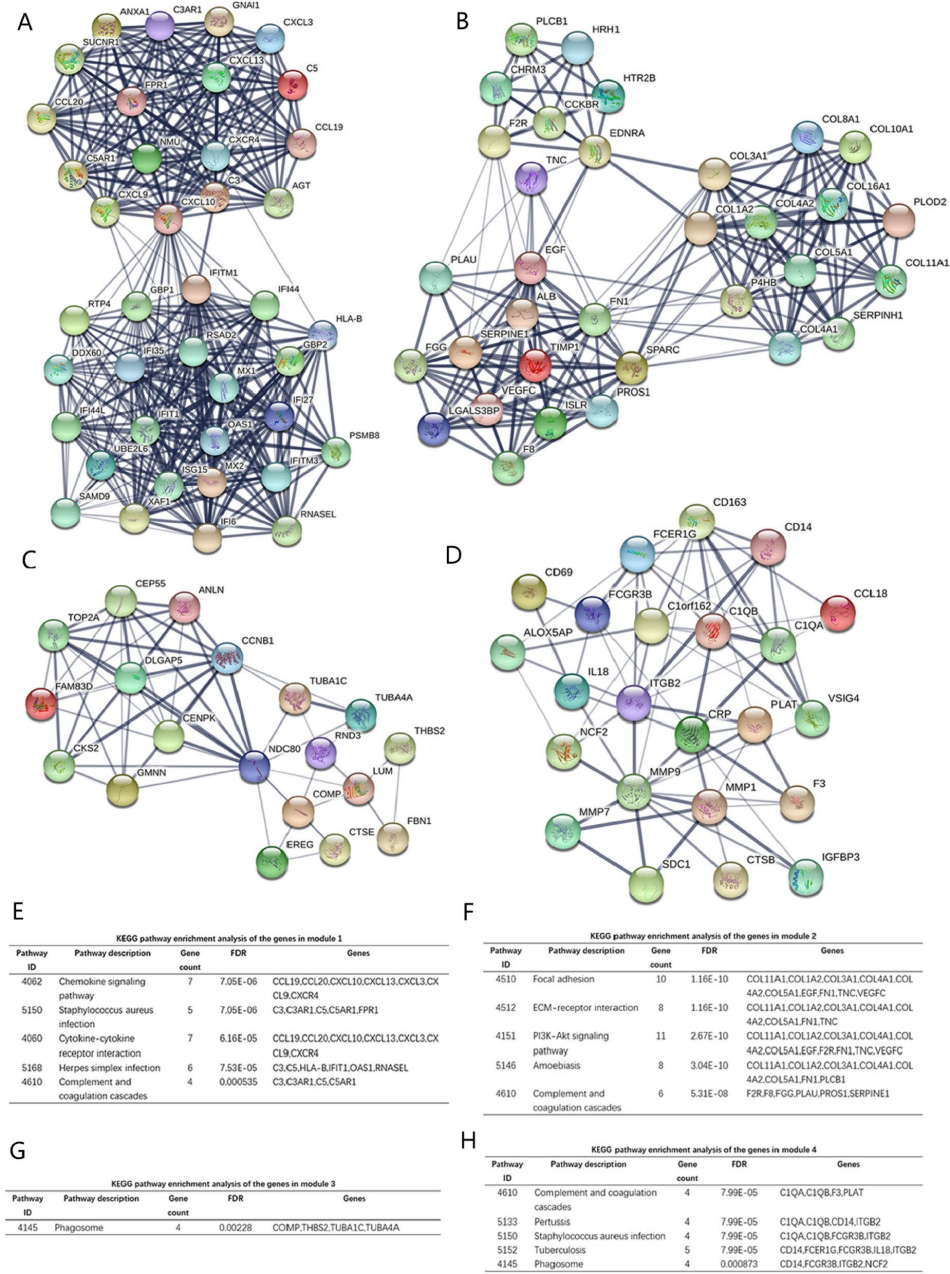
Top four modules from the PPI network. **A** Module 1, **B** module 2, **C** module 3, **D** module 4, **E** the enriched pathways of module 1, **F** the enriched pathways of module 2, **G** the enriched pathways of module 3, **H** the enriched pathways of module 4

According to the STRING database and Cytoscape software [28, 35], the total of 485 differentially expressed genes (362 up-regulated and 123 down-regulated genes) were screened out of the 609 differentially expressed genes to be showed in the PPI network map, including 485 nodes and 2202 edges (Additional file 1: Figure S3). Among the 485 nodes, the top 15 hub nodes with the higher degree of connectivity were identified and shown in Table 3. ALB had the highest node degree of connectivity, which is 120. The PPI network (15 nodes and 42 edges) map of the top 15 key genes with the higher degree were displayed by STRING (Additional file 1: Figure S4). Based on the GO function and KEGG pathway analysis using STRING, we found that COL3A1, EGF, FN1 and ITGA2 were enriched in focal adhesion and PI3K–Akt signaling pathway. Moreover, the whole PPI network was analyzed using Cytoscape MCODE to identify significant modules. Based on the criteria of MCODE score > 4 and number of nodes > 4, the top 4 modules were selected (Table 4). The KEGG pathway enrichment analysis of the genes involved in the 4 modules was calculated by STRING online software (Fig. 3A–D). Pathway enrichment analysis showed that the genes in 4 modules were mainly associated with chemokine signaling pathway, PI3K–Akt signaling pathway, phagosome, tuberculosis (Fig. 3E–H).

**Table 3:** The degree of connectivity of top 15 genes

Gene	Gene name	Degree of connectivity	Expression
ALB	Albumin	120	Down-regulation
EGF	Epidermal growth factor	73	Down-regulation
MMP9	Matrix metallopeptidase 9	53	Up-regulation
CXCL10	C-X-C motif chemokine ligand 10	47	Up-regulation
TOP2A	Topoisomerase (DNA) II alpha	46	Up-regulation
FN1	Fibronectin 1	42	Up-regulation
CTSS	Cathepsin S	40	Up-regulation
ACTB	Actin beta	39	Up-regulation
ITGA2	Integrin subunit alpha 2	38	Up-regulation
ISG15	ISG15 ubiquitin-like modifier	37	Up-regulation
CXCR4	C-X-C motif chemokine receptor 4	37	Up-regulation
OAS1	2ʹ-5ʹ-Oligoadenylate synthetase 1	35	Up-regulation
COL3A1	Collagen type III alpha 1 chain	34	Up-regulation
FPR1	Formyl peptide receptor 1	34	Up-regulation
ITGB2	Integrin subunit beta 2	34	Up-regulation

**Table 4:** Four modules from the PPI network with the criteria of MCODE score > 4 and number of nodes > 4

Cluster	Score	Nodes	Edges	Node IDs
1	19.128	40	373	IFITM1, IFI35, CXCL9, CXCL10, GBP2, FPR1, ANXA1, CCL19, CXCL13, NMU, C5AR1, GNAI1, GBP1, AGT, HLA-B, PSMB8, C3, SAMD9, SUCNR1, MX1, CXCL3, ISG15, IFITM3, IFIT1, OAS1, C3AR1, RSAD2, IFI6, IFI44, IFI27, CCL20, DDX60, MX2, CXCR4, UBE2L6, C5, XAF1, RNASEL, IFI44L, RTP4
2	12.062	33	193	ALB, EGF, HTR2B, VEGFC, CCKBR, PLOD2, HRH1, SERPINE1, EDNRA, PLAU, F8, SERPINH1, COL16A1, TIMP1, PLCB1, P4HB, PROS1, COL11A1, LGALS3BP, ISLR, COL4A1, COL4A2, COL10A1, COL8A1, FGG, TNC, FN1, COL5A1, CHRM3, SPARC, COL3A1, F2R, COL1A2
3	6.556	19	59	COMP, EREG, DLGAP5, TUBA1C, TOP2A, CTSE, LUM, TUBA4A, GMNN, FBN1, NDC80, THBS2, CKS2, CCNB1, ANLN, RND3, CEP55, CENPK, FAM83D
4	6.545	23	72	CD14, ITGB2, SDC1, IGFBP3, CD163, F3, CRP, MMP7, CD69, MMP1, C1orf162, PLAT, NCF2, CCL18, VSIG4, MMP9, CTSB, FCGR3B, FCER1G, C1QA, ALOX5AP, C1QB, IL18

Score = Density*#Nodes

### Comparison of the hub genes expression level

**Fig. 4:**
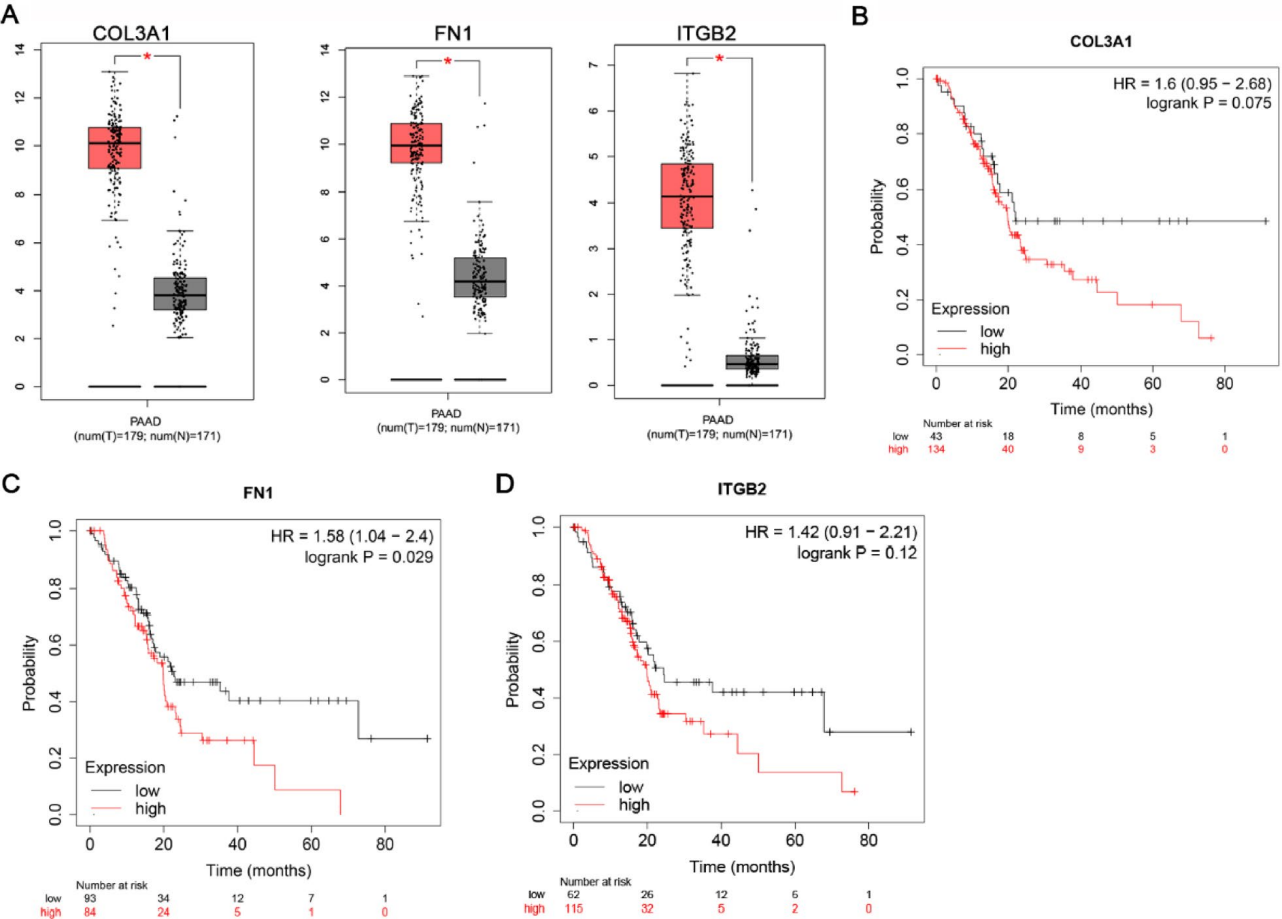
Expression level of hub genes and overall survival in pancreatic cancer and normal tissues. **A** COL3A1, FN1 and ITGB2 level in TCGA and GTEx. *PAAD* Pancreatic adenocarcinoma; *P < 0.01. Red box: tumor; grey box: normal. **B** Overall survival of COL3A1. **C** Overall survival of FN1. **D** Overall survival of ITGB2. n = 177

In order to validate the expression of hub genes between cancer patients and healthy people, we used GEPIA to analyze the data from TCGA normal and GTEx database. Ninety percent of Pancreatic Adenocarcinoma (PAAD) are pancreatic ductal adenocarcinomas (PDAC). Because PAAD is not classified on TCGA website and it can only be analyzed with PAAD data. The data of PAAD were used to indirectly reflect the situation of PDAC. Figure 4A reflected that compared to normal tissue, the hub genes identified in this study were also significantly abnormal expressed in the TCGA and GTEx pancreatic cancers.

We further analyzed the overall survival of COL3A1, FN1 and ITGA2 in pancreatic cancer and normal tissues. As illustrated in Fig. 4B–D, analysis based on transcriptome sequencing data showed that high FN1 expression was significantly associated with low overall survival.

### Profiles of protein expression

**Fig. 5:**
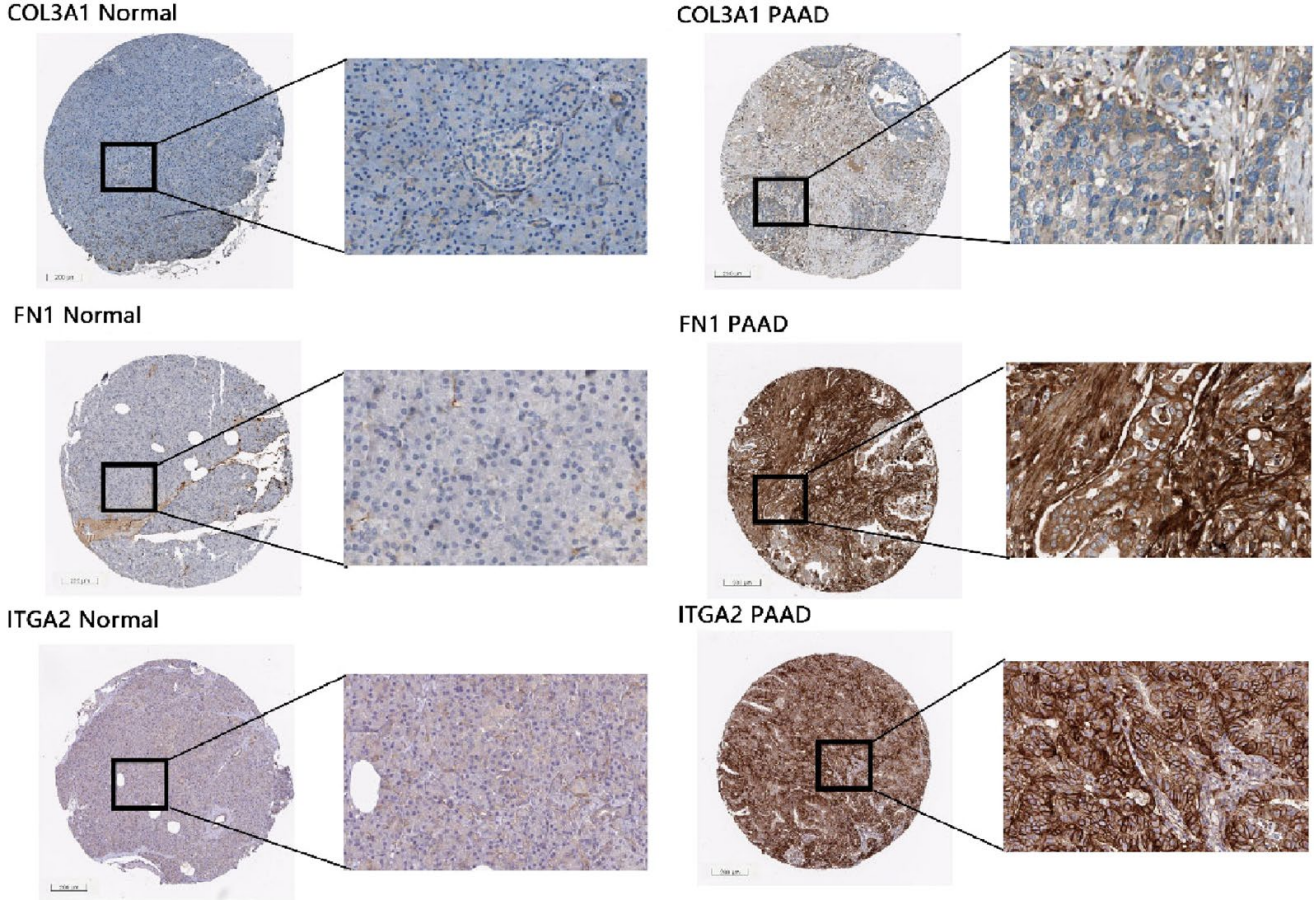
Validation of COL3A1, FN1 and ITGA2 protein level of PI3K–Akt pathway in the HPA database

In addition, the protein levels of COL3A1, FN1 and ITGA2 in pancreatic cancer were improved according to HPA (Fig. 5). Unfortunately, there is no EGF immunohistochemical data. Antibody HPA007583 targeting the COL3A1 protein, HPA027066 targeting the FN1 and HPA063558 targeting the ITGA2 were tested by immunohistochemistry on the normal and PAAD tissue. COL3A1 exhibited medium immunoreactivity in PAAD while staining was low in the normal pancreas; FN1 exhibited medium immunoreactivity in PAAD while staining was not detected in the normal pancreas; ITGA2 exhibited high immunoreactivity in PAAD while staining was low in the normal pancreas.

### Sensitivity of the PI3K–Akt signaling pathway inhibitors to pancreatic cancer

**Fig. 6:**
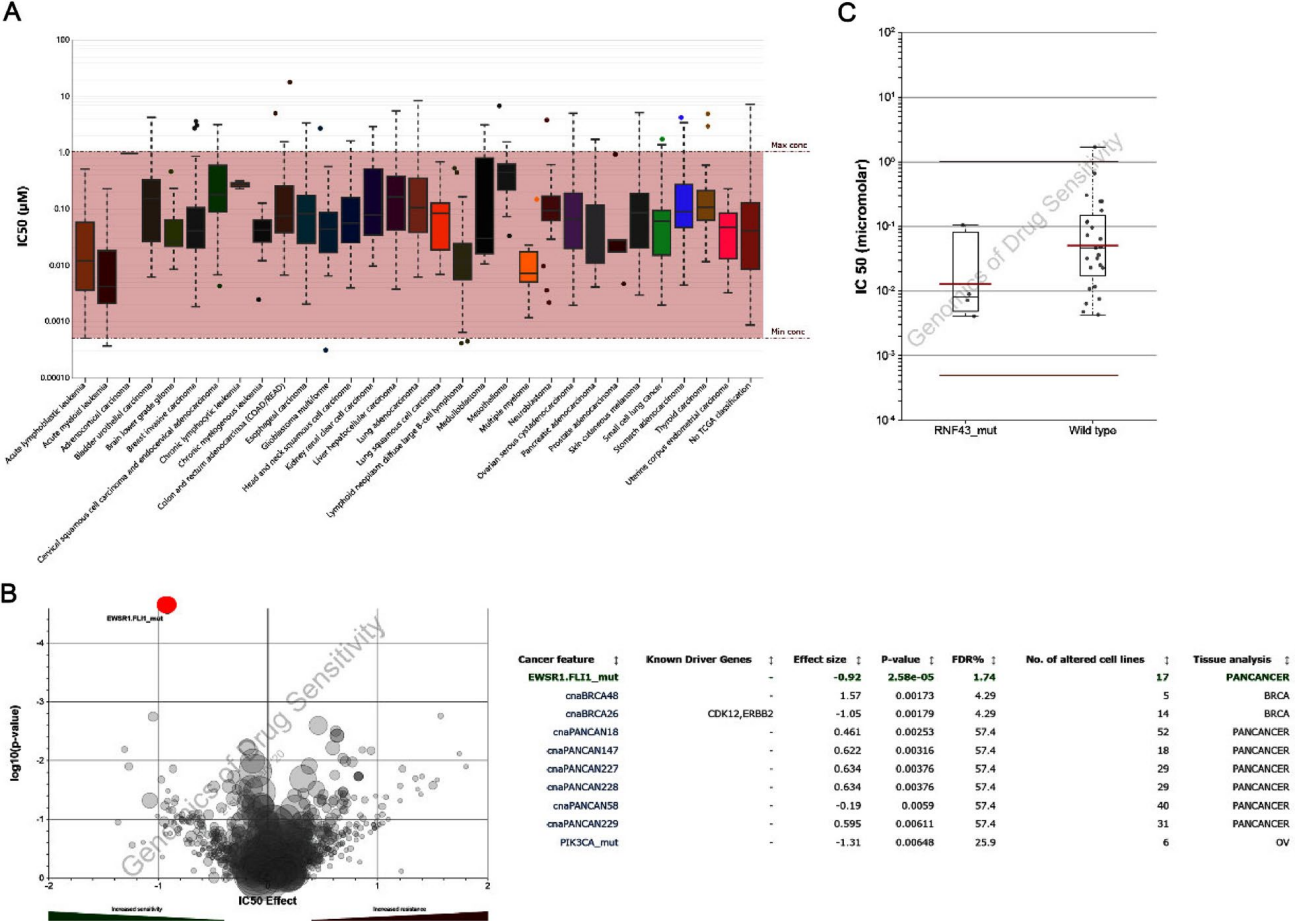
The predictive effects of mTOR inhibitor-GSK2126458 on cancer cells. **A** IC_50_ distribution of GSK2126458 by tissue type. **B** Volcano map of sensitivity of GSK2126458 to cancer cell lines. **C** IC_50_ of GSK2126458 on pancreatic cancer gene mutant cell line

Most of these key genes are enriched in PI3K–Akt pathway, we investigated the effect of three PI3K–Akt signaling pathway inhibitors on the cell growth of PANC-1 (human pancreatic cancer cell lines). BKM-120 is a selective PI3K inhibitor; MK2206 is a highly selective Akt1/2/3 inhibitor and the first small molecule Akt allosteric inhibitor to enter clinical research; GSK2126458 is a highly selective, potent p110α/β/γ/δ and mTORC1/2 inhibitor. These three compounds are inhibitors of three key targets in the PI3K–Akt–mTOR signaling pathway, respectively. The IC_50_ values on pan-cancer cell lines of BNM-120, MK2206 and GSK2126458 were predicted with the GDSC data, and our analysis indicated GSK2126458 with a significant response sensitivity against pancreatic cancer cell lines (Fig. 6A). As shown in Fig. 6B, EWSR1-FLI1 mutant pancreatic cell was the most sensitive to GSK2126458. In addition, the sensitivity of GSK2126458 to FNR43 mutant pancreatic cancer cells was significantly higher than that of wild-type cells (Fig. 6C).

### The effects of mTOR inhibitor-GSK2126458 on pancreatic cancer cells

**Fig. 7:**
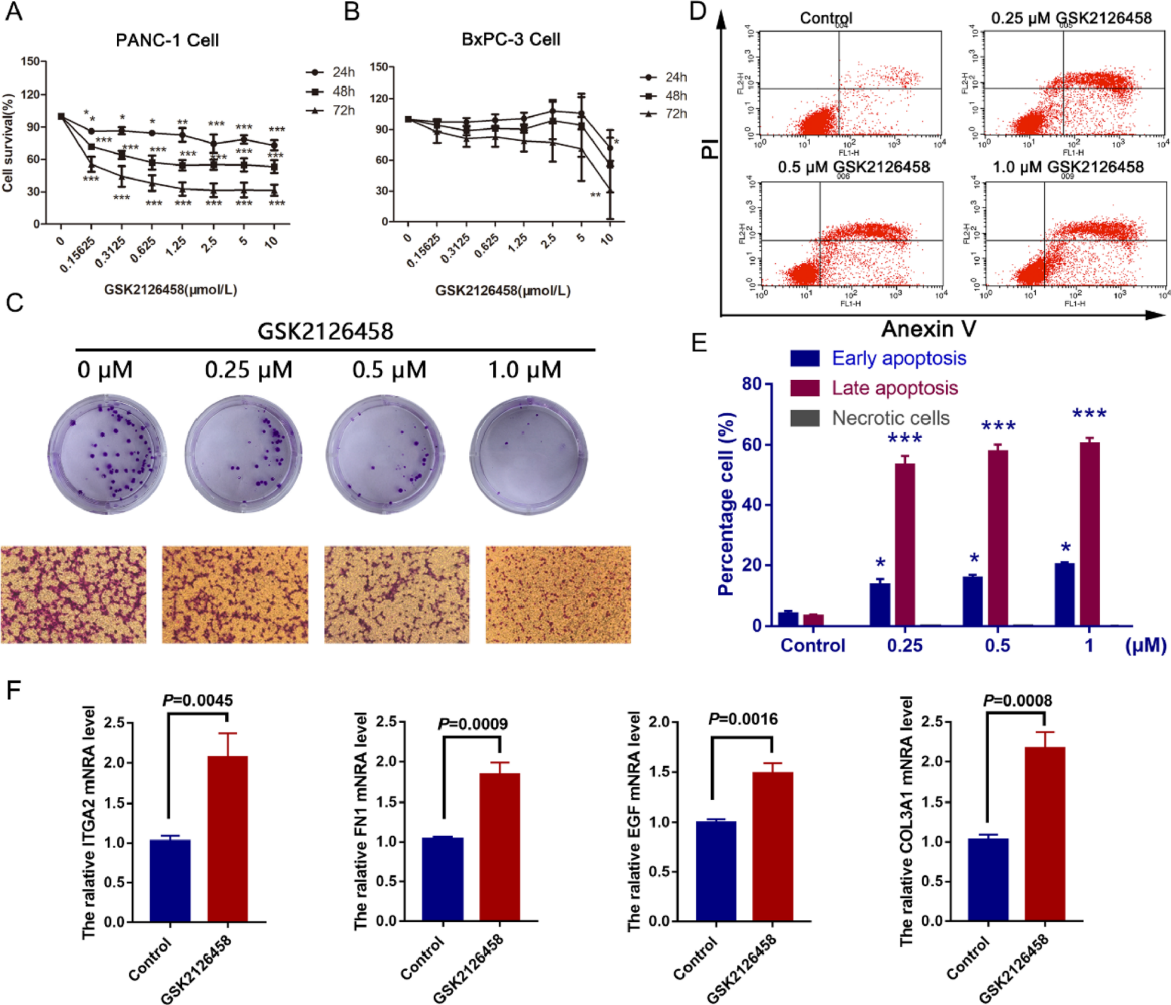
The effects of mTOR inhibitor-GSK2126458 on pancreatic cancer cells. **A** MTT result of GSK216458 on PANC1 cell. **B** MTT result of GSK216458 on BxPC-3 cell. **C** Colony formation invasion results of GSK216458 on PANC1 cell. **D** FACS result of GSK216458 on PANC1 cell. **E** Statistical histogram of FACS results. **F** The mRNA expression of COL3A1, EGF, FN1 and ITGA2. The data came from three independent experiments. *P < 0.05, **P < 0.01, ***P < 0.001, compared with the control group

As illustrated in Fig. 7A, GSK2126458 decreased the cell viability of PANC-1 cells in time and dose-dependent manners after 24–72 h treatment. The IC_50_ of GSK216458 on PANC-1 cells were > 10 μM for 24 h, 0.87 ± 0.17 μM for 48 h, 0.23 ± 0.13 μM, respectively. An ideal therapeutic drug of pancreatic cancer is the one that can inhibit cancer cell growth but has no toxic effect to normal tissue. Therefore, we investigated the effect of GSK2126458 on HL7702 cell. We found that the GSK2126458 (1 μM) had no significant effect on the cell survival rate of HL7702 cell (Additional file 1: Figure S6). After treatment with gsk216458 for 48 h, the colony formation of PANC-1 cells was significantly inhibited in a dose-dependent manner (Fig. 7C). In addition, we also investigated the effect of GSK216458 on the invasion of PANC-1 cells. As shown in Fig. 7C, GSK216458 can significantly suppress the invasion of PANC-1 cells. FACS results showed that GSK2126458 could significantly induce the late apoptosis of PANC-1 cells (Fig. 7E, F). FACS results of BxPC-3 cells was seen in Additional file 1: Figure S7. These results showed that GSK2126458 can inhibit the proliferation and apoptosis of pancreatic cancer cells in a time- and dose-dependent manner.

## Discussion

Compared with other malignant tumors, the survival rate of advanced PDAC is the lowest, with a median overall survival of 2–8 months and a 5-year survival rate of 8.5%. This is due to the lack of effective screening tools, most of which are advanced (~ 80%) and the limited effect of FDA approved drugs [36]. Monotherapy with fluoropyrimidine, gemcitabine, irinotecan, platinum and Taxane in patients with advanced PDAC has low remission rate and poor survival benefit, and multi drug combination therapy provides slightly higher response rate and slightly higher survival benefit [4]. Therefore, our study intends to provide a new scheme for the treatment of PDAC through bioinformatics technology.

In the current study, a total of 432 up-regulated and 177 down-regulated genes were screened out by analyzing two gene expression profiles. These results suggested that the common DEGs may play a key role in the development of pancreatic cancer. We investigated the potential role of DEGs using functional enrichment analysis. Firstly, the GO functional enrichment revealed that the function of up-regulated genes enriched in areas such as extracellular matrix organization with the high significant P-value. Extracellular matrix is a dynamic microenvironment in the development of cancer [37–40]. Specifically, one of the main characteristics of pancreatic cancer is the rich extracellular matrix, which plays a vital role in the growth and metastasis of tumor cells [41]. Molecular markers such as COL11A1, SERPINE1, TGFBI, TNC and LUM were enriched in the extracellular matrix organization. Among them, the COL11A1 gene is overexpressed in pancreatic cancer and the protein encoded by the COL11A1 gene may be involved in fibrous proliferative events in pancreatic cancer [42]. LUM regulates collagen fibrillogenesis in the extracellular matrix. Recently, LUM has been reported to regulate cell behavior during embryonic development, tissue repair and tumor progression [43, 44]. These findings suggested that the microenvironment, especially the abnormality of the extracellular matrix, plays a key role in the development of pancreatic cancer.

Similarly, GO functional enrichment showed that down-regulated genes were mainly involved in reactive oxygen species metabolic process with the high significant P-value. It was reported that some factors associated with the increasing incidence of pancreatic cancer are also factors that lead to reactive oxygen species (ROS) overexpression [45]. For pancreatic cancer, ROS is a double-edged sword based on the concentration of intracellular ROS. At mild to moderate levels, ROS contributes to the proliferation, metastasis and invasion of tumor cells.

After analysis, we found that the above KEGG pathway is not related to the mechanism of tolerance to starvation in pancreatic cancer. Then we analyze the signal pathways with more enriched genes. The three KEGG pathways that enrich most genes are focal adhesion, phagosome and PI3K–Akt signaling pathway. Focal adhesion kinase, a member of the FAK subfamily, is mainly found in the focal adhesion signaling pathway. Previous studies have shown that inhibition of FAK signaling helps to suppress various types of cancer, including non-small cell lung cancer, breast cancer, ovarian cancer, etc. [46–48]. Phosphorylated FAK can activate or inhibit several downstream pathways, including PI3K/Akt and p53, which initiate tumorigenesis or induce apoptosis [49]. All these are signal pathways which are closely related to the occurrence and development of tumors. Tumor phagosome signaling pathway is mainly autophagy, which is a double-edged sword in cancer treatment [50]. Although these two over-expressed signaling pathways are inextricably linked to the development of pancreatic cancer, there is no better explanation for the specific mechanisms by which pancreatic cancers can be high tolerance of hypoxic and nutrient-deficient environments. Finally, we focused on the PI3K–Akt signaling pathway.

Akt is a key regulator of cell proliferation and growth, which is often overexpressed in cancer cells [51, 52]. As we all known, the lack of blood supply in pancreatic cancer leads to the limited availability of nutrients and oxygen to the tumor cells [53, 54]. For further verification, we built the PPI network with 609 DEGs and screened the top 15 hub genes with the high degree: ALB, EGF, MMP9, CXCL10, TOP2A, FN1, CTSS, ACTB, ITGA2, ISG15, CXCR4, OAS1, COL3A1, FPR1 and ITGB2. Of these, ALB and EGF were down-regulated and the others were all up-regulated. Among them, ALB was the highest degree of connectivity gene in the hub genes. Then we used GEPIA to estimate the expression level of hub genes in cancer and normal tissues. As shown in the box plots, these results were consistent with the trend of expressions of hub genes that we identified involved in the PPI network. It further demonstrated that the results of the hub genes we have identified are reliable. It is noticeable that 4 (COL3A1, EGF, FN1 and ITGA2) of the top 15 highly expressed genes were key proteins in the PI3K–Akt signaling pathway. After the module analysis of the PPI network, 4 significant modules were selected and then the pathway enrichment analysis was carried out. Here we continue to notice the PI3K–Akt signaling pathway, which involves the largest number of genes. The PI3K–Akt signaling pathway in module 2 contains 11 genes, of which 2 (COL3A1 and FN1) belong to the hub genes. All these suggested that the PI3K–Akt signaling pathway plays a key role in the development of pancreatic cancer, which is consistent with the previous literature. They suggested that about 50% of pancreatic cancers exhibit increased activation of PI3K signaling, which is usually associated with the undifferentiated state of the tumor and poor prognosis [55–57].

In pre-clinical studies of pancreatic cancer, many mTOR inhibitors have shown a variety of inhibitory effects on pancreatic cancer cells and inhibit epithelial to mesenchymal transition, including the first generation of mTOR inhibitors rapamycin, the second generation of mTOR inhibitors such as KU63794 and PP242, and the new mTOR inhibitor INK-128 dual mTOR inhibition [58, 59]. In addition, there are currently 22 clinical trials using mTOR inhibitors to treat pancreatic cancer (https://clinicaltrials.gov). Therefore, the prospect of mTOR inhibitors in the treatment of pancreatic cancer is optimistic.

However, preclinical studies have shown that the combination of mTOR inhibitors and drugs targeting the emergency drug resistance pathway provides a strong theoretical basis for PDAC treatment, it is regrettable that clinical trials have failed to achieve this expected effect. Failure to obtain a meaningful response is multifactorial, due to the reactivation of upstream RTK-driven pathways, the poor vascularization caused by significant interstitial fibrosis, and the toxicity that limits the optimal biological dosing treatment [60].

This is the first report on the anti pancreatic cancer effect of GSK2126458 through bioinformatics and in vitro experiments. There are still many problems to be solved in our research, especially the essential problem in vivo research: the toxicity of limiting the optimal biological dose.

## Conclusion

In summary, our study provided a comprehensive bioinformatics analysis to identify the DEGs which may be involved in the progression of pancreatic cancer. In this study, we screened 609 DEGs and then found 15 significantly changed hub genes from them which mainly related to PI3K–Akt signaling pathway. In addition, we used the GDSC database analysis to prove that the mTOR inhibitor (GSK2126458) is very sensitive to pancreatic cancer cell lines, especially for EWSR1.FLI2 and RNF43 mutant. This is the first time that we report that GSK2126458 has a potential role in the treatment of pancreatic cancer. We will explore the role of GSK2126458 against pancreatic cancer through systematic studies in vitro and in vivo, in order to provide a new strategy for the treatment of pancreatic cancer.

## Supplementary Information

**Additional file 1: Figure S1.** Normalized expression value data box plots. Black line in each box represents the median of each sample. All the black lines are almost in the same position, which indicates high degree of standardization (“the black lines” refers to “median-centered values”). **Figure S2. **Heat maps of the top 50 differentially expressed genes (30 up-regulated and 20 down-regulated) of GSE15471 (A) and GSE62165 (B). Gray, case group; yellow, control group. Red: high expression level; blue: low expression level. **Figure S3.** The PPI network of 485 DEGs (Pink: 362 up-regulated genes; blue: 123 down-regulated genes) which were screened out by STRING. **Figure S4.** The PPI network of top 15 hub genes. **Figure S5.** Expression level of hub genes and overall survival in pancreatic cancer and normal tissues. **Figure S6.** MTT result of GSK216458 on HL7702 cells. **Figure S7.** FACS results of GSK216458 on GxPC-3 cells. **Table S1.** 609 DEGs were identified from two profile datasets.

## Data Availability

All data generated or analysed during this study are included in this published article [and its additional information files].

## Abbreviations

GEO: Gene Expression Omnibus
DEGs: Differentially expressed genes
GO: Gene ontology
KEGG: Kyoto Encyclopedia of Genes and Genomes
DAVID: The Database for Annotation, Visualization and Integrated Discovery
STRING: Search Tool for the Retrieval of Interacting Genes
PPI: Protein–protein interaction
BP: Biological process
CC: Cellular component
MF: Molecular function
MCODE: Molecular Complex Detection
TCGA: The Cancer Genome Atlas
GTEx: Genotype-Tissue Expression
GEPIA: Gene Expression Profiling Interactive Analysis
COL3A1: Collagen type III alpha 1 chain
EGF: Epidermal growth factor
FN1: Fibronectin 1
ITGA2: Integrin subunit alpha 2
ALB: Albumin
MMP9: Matrix metallopeptidase 9
CXCL10: C-X-C motif chemokine ligand 10
TOP2A: Topoisomerase (DNA) II alpha
CTSS: Cathepsin S
ACTB: Actin beta
ISG15: ISG15 ubiquitin-like modifier
CXCR4: C-X-C motif chemokine receptor 4
OAS1: 2ʹ-5ʹ-Oligoadenylate synthetase 1
FPR1: Formyl peptide receptor 1
ITGB2: Integrin subunit beta 2
PI3K: Phosphatidylinositol-3-OH kinase
Akt: Protein kinase B identified in the Akt virus
GDSC: The Genomics of Drug Sensitivity in Cancer Project
MTT: 3-(4,5-Dimethylthiazol-2-yl)-2,5-diphenyltetrazolium bromide
DMSO: Dimethyl sulfoxide
