# Supplementary material for: GSK2126458 has the potential to inhibit the proliferation of pancreatic cancer uncovered by bioinformatics analysis and pharmacological experiments
Source: J Transl Med. 2021 Aug 30;19:373. doi: 10.1186/s12967-021-03050-7 (PMC8406597; doi:10.1186/s12967-021-03050-7)
Supplement: Supplementary file 1 — Additional file 1: Figure S1. Normalized expression value data box plots. Black line in each box represents the median of each sample. All the black lines are almost in the same position, which indicates high degree of standardization (“the black lines” refers to “median-centered values”). Figure S2. Heat maps of the top 50 differentially expressed genes (30 up-regulated and 20 down-regulated) of GSE15471 (A) and GSE62165 (B). Gray, case group; yellow, control group. Red: high expression level; blue: low expression level. Figure S3. The PPI network of 485 DEGs (Pink: 362 up-regulated genes; blue: 123 down-regulated genes) which were screened out by STRING. Figure S4. The PPI network of top 15 hub genes. Figure S5. Expression level of hub genes and overall survival in pancreatic cancer and normal tissues. Figure S6. MTT result of GSK216458 on HL7702 cells. Figure S7. FACS results of GSK216458 on GxPC-3 cells. Table S1. 609 DEGs were identified from two profile datasets. [file 12967_2021_3050_MOESM1_ESM.docx]

**Additional file**

**
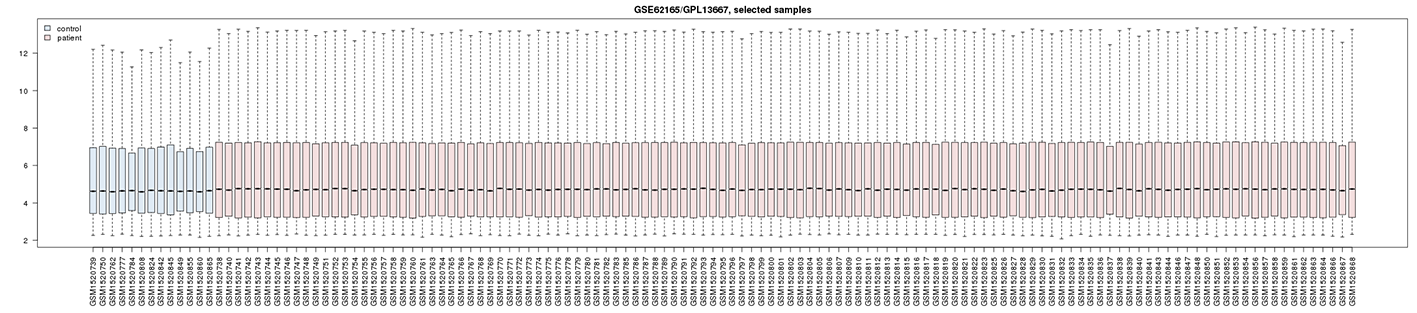

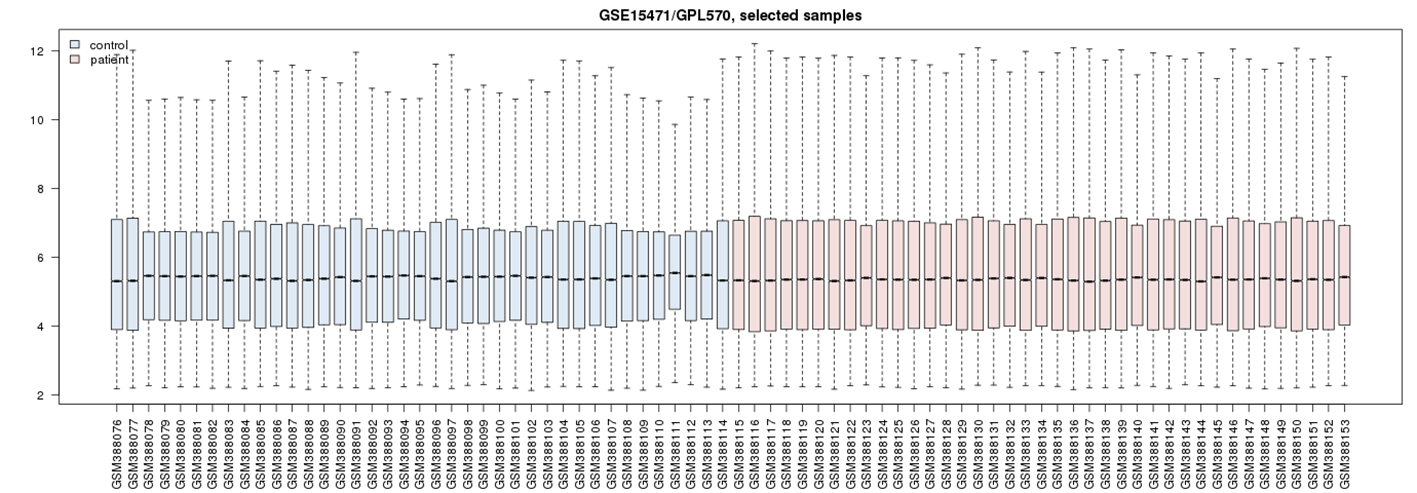
**

**Figure S1.** **Normalized expression value data box plots. Black line in each box represents the median of each sample. All the black lines are almost in the same position, which indicates high degree of standardization (“the black lines” refers to “median-centered values”).**

**
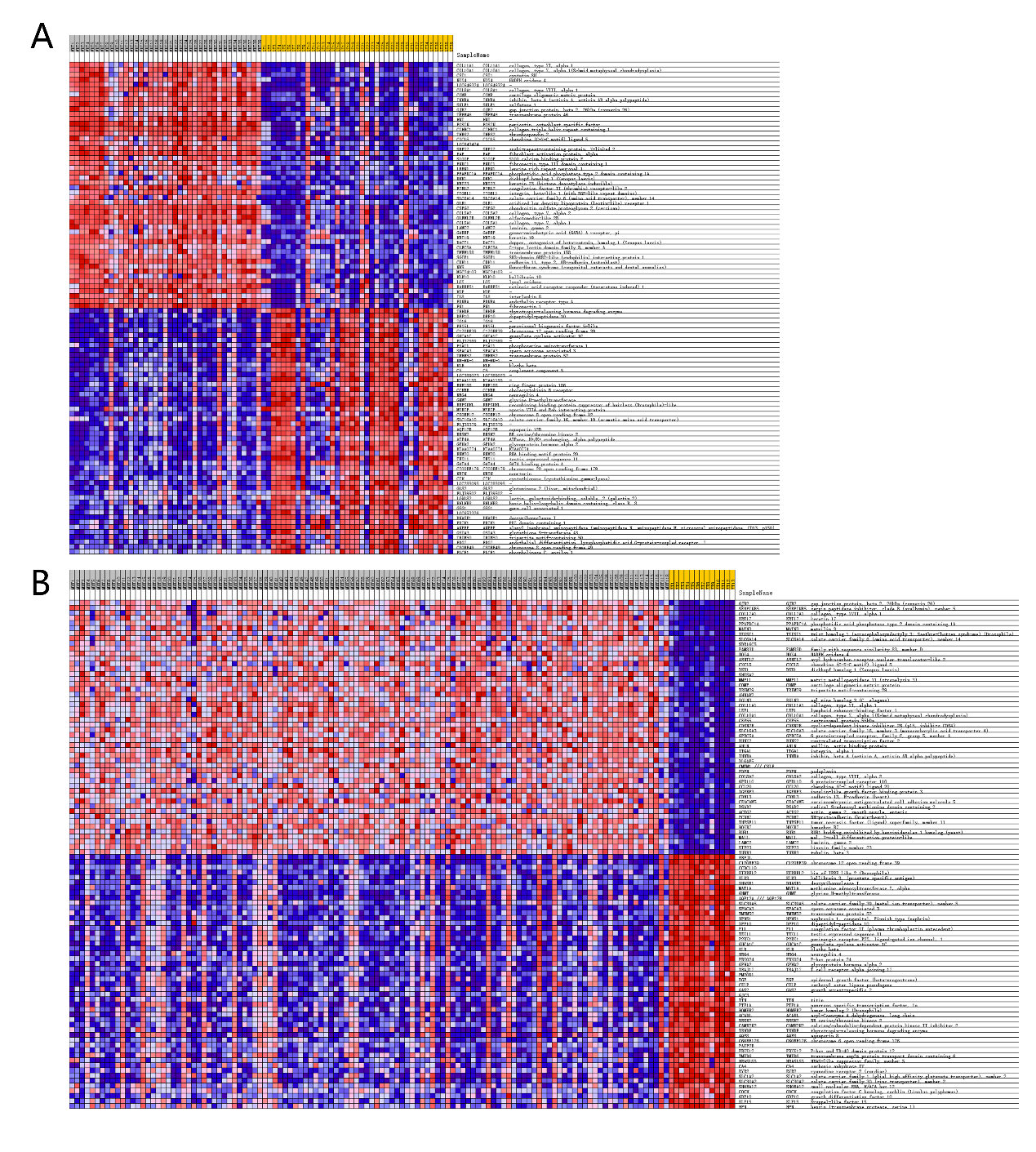
**

**Figure S2.** Heat maps of the top 50 differentially expressed genes (30 up-regulated and 20 down-regulated) of GSE15471 (A) and GSE62165 (B). Gray, case group; yellow, control group. Red: high expression level; blue: low expression level.

**
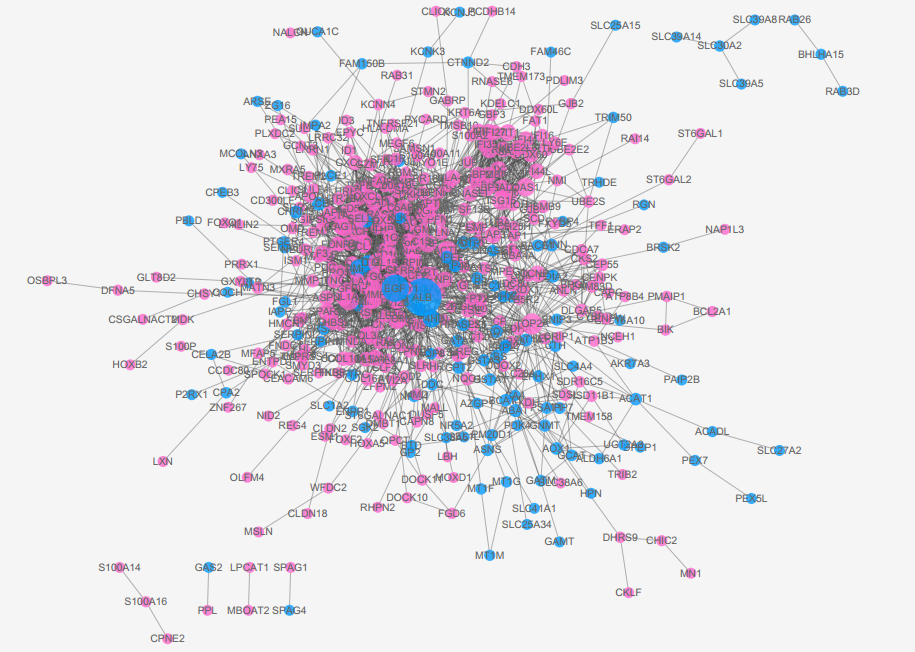
**

Figure S3. The PPI network of 485 DEGs (Pink: 362 up-regulated genes; blue: 123 down-regulated genes) which were screened out by STRING.

**
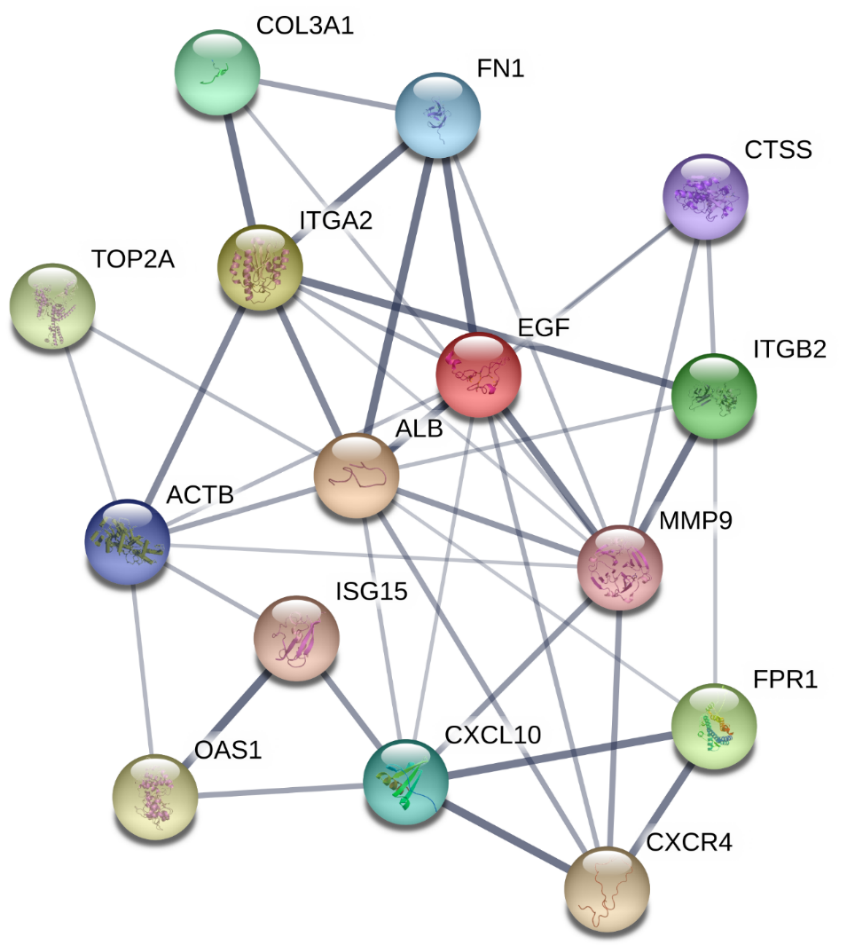
**

Figure S4. The PPI network of top 15 hub genes.

**
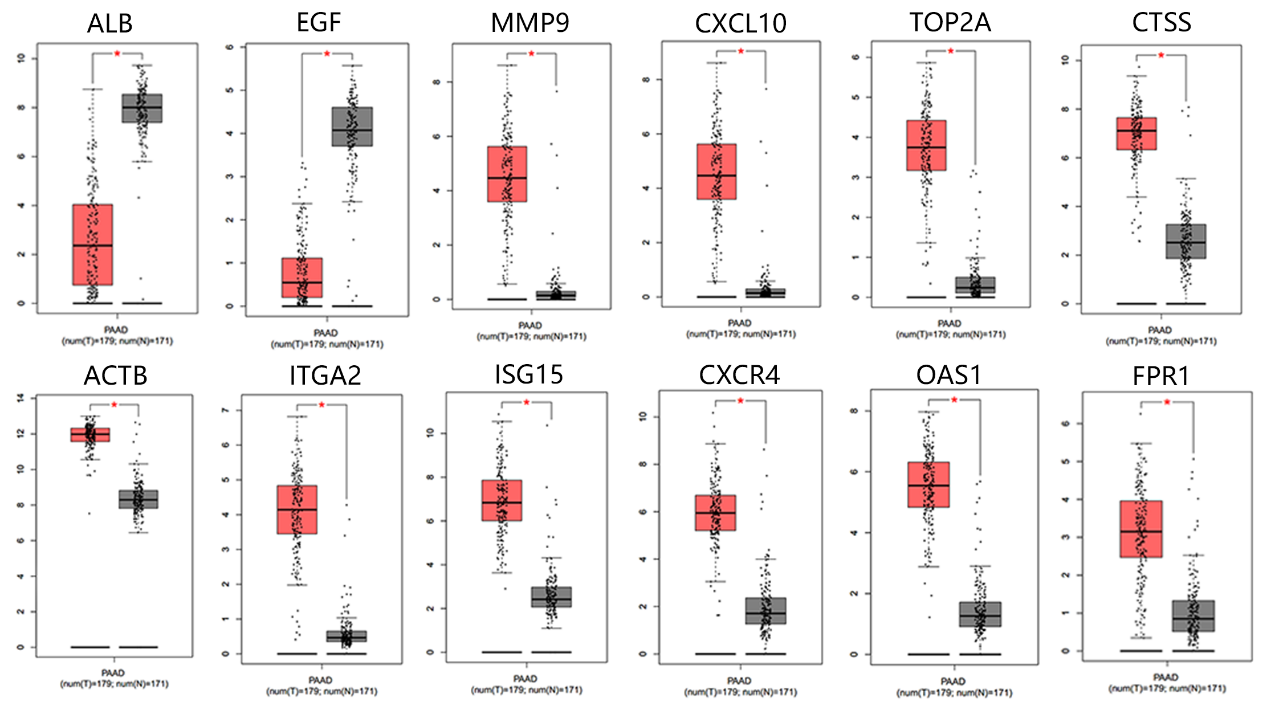
**

Figure S5. Expression level of hub genes and overall survival in pancreatic cancer and normal tissues.


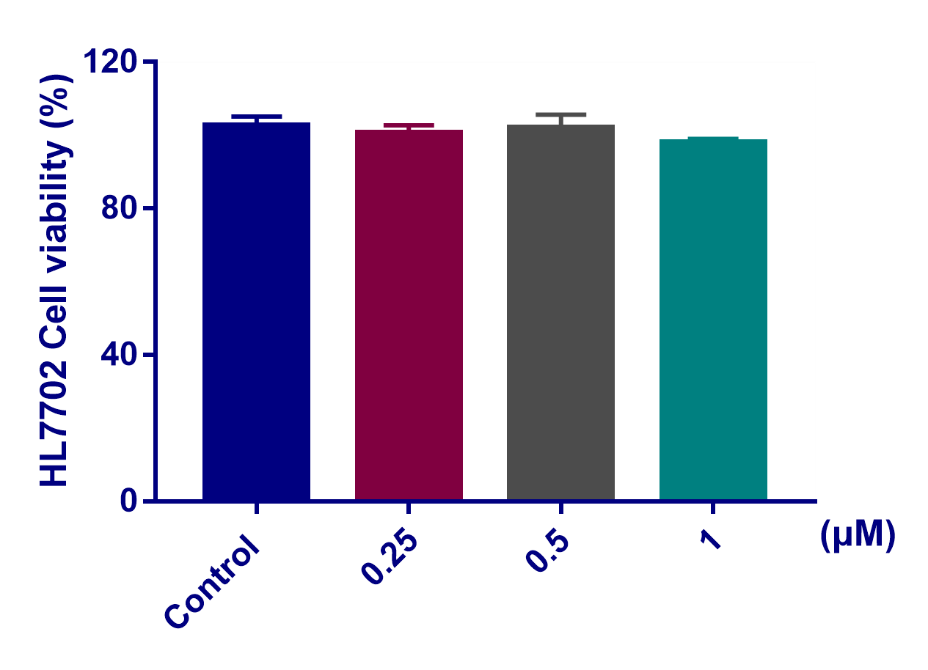


Figure S6. MTT result of GSK216458 on HL7702 cells.


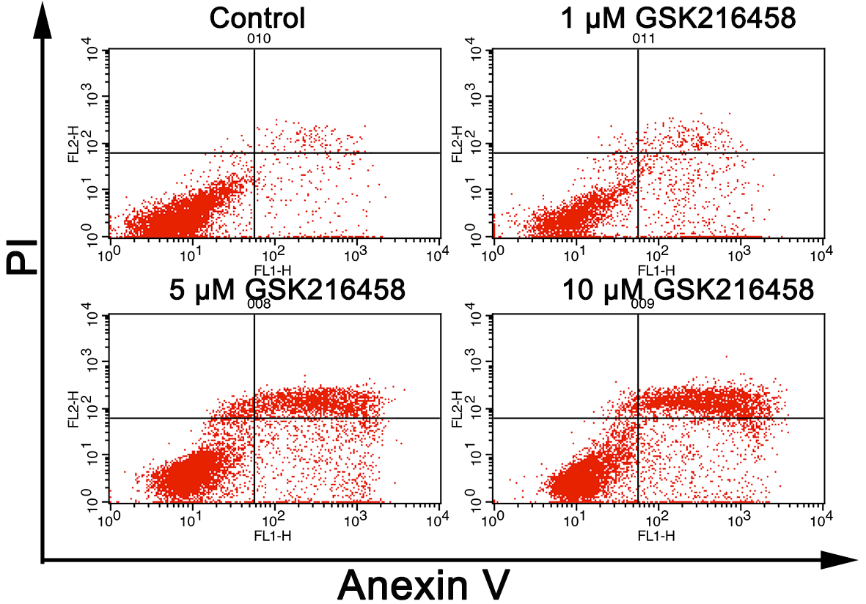


Figure S7. FACS results of GSK216458 on GxPC-3 cells.

| Table S1. 609 DEGs were identified from two profile datasets. | | |
| --- | --- | --- |
| DEGs | | Genes Name |
| Up-regulated | PNMA1, XDH, CXCR4, TMSB10, PLAU, CXCL3, ANXA1, C1R, MMP7, ID3, S100P, C1QC, CILP, C15orf48, CCNB1, FOXQ1, SLC16A14, HEPH, CEACAM6, AGR3, SFTA2, LRRN1, CXCL13, BIK,  SH3RF3, TRIM22, RNASE6, CHSY1, EPS8, GPNMB, ITGA3, PTPN12, GPR137B, C5AR1, GLT8D2, ANXA3, RAC2, CD55, CSGALNACT2, ANLN, MRVI1, SMYD3, TMEM45A, GJB6, SELL, GFPT2,  CCL19, EVI2A, ISM1, LGALS1, C3, FGG, FNDC1, C3AR1, TNFRSF11B, SAMSN1, EMILIN1, DUSP5, ID1, PXDN, NCF2, EDNRA, TMEM200A, RHOH, RAI14, HMCN1, CLDN2, LTBP1, DUOX2, CFH,  TAP1, PRDX1, AGT, SIPA1L2, SGIP1, MFAP5, NCEH1, KDELC1, CD53, CRP, CST1, UBE2L6, PRRG1, DOCK11, SLC12A2, MNDA, ANXA2P2, FN1, AHR, CPNE2, LOXL1, PRRX1, IFI27, IFI16, CSTA,  ASAP2, LTBP2, SFRP2, NUAK1, PPL, LAMB3, TNFAIP6, SERPINB2, ZFPM2, IER3, OMD, CTSB, CTSS, OAS1, TMEM47, ATP8B4, DPCR1, PLS1, LXN, NALCN, RAB23, FKBP10, VEGFC, FHL2, LGALS3BP,  S100A14, THY1, DDX60, C5orf46, LDHA, SCD, OSBPL3, TMPRSS4, CORO1C, CAPG, CCL20, XAF1, SDR16C5, WNT5A, MATN3, LGALS4, CAP1, SLPI, HOXA5, IER5L, GPRC5A, DRAM1, ELF4, GBP2,  IL18, CTSK, SULF2, RRAS, EREG, SFRP4, VSIG4, CDCA7, SFN, MXRA5, RND3, GPX2, ITGB2, RCN1, GXYLT2, PROS1, PSMB8, HN1, NDC80, ITGA2, S100A6, QPCT, PELI1, MALL, S100A16, FBN1, PLOD2,  RAB31, PI3, ZG16B, SLC6A14, KRT6A, ZNF267, CLIC1, MMP1, S100A11, DFNA5, COL8A1, MMP12, COLEC12, MEGF6, MVP, BCL2A1, CKS2, ISG15, DHRS9, MX2, SLC38A6, FPR1, ASPN, MYO1E, CD14,  CAPN8, CDH3, TAGLN, COL3A1, CD163, GIMAP2, COL4A2, LUM, C1S, BPGM, TUBA4A, MOXD1, ERAP2, TMEM45B, TYROBP, TMEM158, TNC, LRRC32, IFITM3, EMB, CCL18, SERPINH1, UBE2S,  PCDHB14, MN1, FAP, SGK223, HTR2B, PLEK2, NAP1L3, INHBA, IFI6, S100A4, FAM198B, ANXA10, LGMN, TCN1, GPR183, ISLR, ALOX5AP, DNMT1, PLXDC2, OLFM4, REG4, GZMA, TCEAL7, TREM2,  PLAT, ITGBL1, PEA15, LY75, GNAI1, NQO1, THBS4, DMBT1, FXYD5, SRPX2, CKLF, SDC1, IFI44L, IFIT1, TNFSF13B, CENPW, CCDC80, GBP1, DOCK10, CEP55, IGDCC4, LEMD1, GPR34, CTSE, TMEM173,  EPYC, MDK, KLF5, EVI2B, APOD, GABRP, TOP2A, SLC20A1, EMP3, GJB2, COMP, EFNB2, CXCL9, F2R, EGFL6, RNASEL, TUBA1C, AEBP1, SLC16A4, TNFRSF21, FCGR3B, CHIC2, THBS2, FOXF1, PMAIP1,  S100A2, STEAP2, NID2, HRH1, ST6GAL2, C19orf33, BTN3A3, C1QB, HTRA1, BHLHE40, RTP4, WFDC2, COL16A1, GPX8, TMEM200B, DKK1, OLFML2B, S100A10, MAFB, DLGAP5, BAZ1A, C1orf54, IL7R,  ACTB, TSPAN1, NMI, IFI35, CYB5R2, KRT19, ENO1, CXCL10, ZNF532, CRIP1, SLC7A7, SERPINE1, COL4A1, SLFN11, GBP3, FAM83D, SPAG1, EMILIN2, ENTPD1, RSAD2, JUP, MX1, GLIPR1, HLA-DMA,  MSLN, SULF1, DDX60L, UBE2E2, HLA-DRB1, MMD, HLA-B, CD300LF, LAMC2, LY6E, LYZ, RARRES1, FAT1, LAPTM5, GCNT3, MPEG1, RHPN2, PDLIM3, LAP3, NMU, MXD1, PON2, LMCD1, PFN1, CENPK,  NEURL1B, CTSA, IGSF6, COL10A1, ST6GAL1, FCER1G, SLC24A3, TGFB1I1, SAMD9, HSD11B1, LCN2, SPARC, GPR87, IGFBP3, COL5A1, PLAC8, C1QA, TPST1, PMEPA1, F3, COL11A1, FOXF2, TREM1,  PRICKLE1, CD69, SUCNR1, GPX1, SERPINB3, AHNAK2, IFITM1, GEM, STMN2, CYBRD1, MMP10, NPL, LBH, TFF1, TGFBI, SNAI2, ATP1B3, KCNN4, HOXB2, E2F3, CLDN18, FGD6, LPCAT1, FLNA,  TWIST1, COL1A2, CP, LY96, TRIB2, PYCARD, MBOAT2, CLIC6, C1orf162, PDGFC, CH25H, IFI44, RBMS1, KRT23, PSMB9, ST6GALNAC1, MMP9, SPOCK1, SLC44A4, ESM1 | |
| Down-regulated | PLCE1, DNASE1, BTG2, HPN, GSTA1, CTNND2, EPB41L4B, PEBP4, CCDC110, RGN, ZG16, SLC38A5, KCNK3, VEPH1, FAM129A, SLC39A14, A1CF, ACAT1, TMEM52, MPP7, FAM150B, ATP4A, BANF2, CPEB3, KIAA1324, SEC11C, ACADL, PRDX4, SPACA3, RAB26, MT1M, SLC39A5, AKR7A3, PBLD, NRG4, PXMP2, CUZD1, F11, MT1G, CPA2, BNIP3, ALDH6A1, C5, EGF, FAM3B, AQP8, SLC1A2, RBM20, PEX7, TDH, SLC25A34, CBS, GAMT, PAK3, SGK2, IGFBP2, IMPA2, CTH, SLC17A4, UGT2A3, ECHDC3, HRASLS5, TRHDE, CCKBR, PRSS3, CRAT, GP2, TPST2, SPAG4, BHLHA15, COCH, SLC30A2, F8, GPHA2, PDK4, RBPJL, NRTN, EPHX2, REEP1, PTGER4, SLC25A15, PNLIPRP2, SEL1L, LGALS2, MTUS2, PDIA2, PM20D1, GIPC2, BACE1, CA4, PLCB1, NUCB2, IAPP, HOMER2, SERPINI1, LIFR, RAB3D, PSAT1, ERP27, MCOLN3, KIRREL2, AZGP1, SLC43A1, GNMT, CELP, EPHX1, CELA2B, NR5A2, ABAT, KLB, BRSK2, SEMA6D, PIWIL2, ASNS, IL22RA1, GSTA3, TMEM97, SIDT2, DDC, ANPEP, PRLR, P2RX1, GAS2, PAIP2B, DPEP1, SYBU, MAT1A, RNASE1, LMO3, SLC39A8, CCDC69, SFTPC, RNF186, MT1F, SLC41A1, FGL1, GUCA1C, CTRL, SMPDL3B, MYRIP, GCAT, ENPP1, SYCN, DMD, GMNN, PNLIPRP1, KLK1, SLC27A2, TCEA3, FAM46C, ARSE, CHRM3, SLC25A45, P4HB, STC2, BTD, TMED6, ALB, GPT2, KCNJ5, CYB5A, BSPRY, PEX5L, BCAT1, KCNJ16, SERPINI2, GATM, TEX11, FKBP11, SLC4A4, DPP10, AOX1, GLS2, GATA4, SLC16A10, TRIM50, CBFA2T3 | |
